# Oropharyngeal Microbiome Analysis in Patients with Varying SARS-CoV-2 Infection Severity: A Prospective Cohort Study

**DOI:** 10.3390/jpm14040369

**Published:** 2024-03-29

**Authors:** Panagiotis Siasios, Evangelia Giosi, Konstantinos Ouranos, Maria Christoforidi, Ifigenia Dimopoulou, Enada Leshi, Maria Exindari, Cleo Anastassopoulou, Georgia Gioula

**Affiliations:** 1 Microbiology Department, School of Medicine, Aristotle University of Thessaloniki, 54124 Thessaloniki, Greece; psiasio@auth.gr (P.S.); egiosi@auth.gr (E.G.); mhrist@auth.gr (M.C.); ifigeniadim@auth.gr (I.D.); eleshi@auth.gr (E.L.); mexidari@auth.gr (M.E.); ggioula@auth.gr (G.G.); 2 Department of Medicine, Houston Methodist Research Institute, Houston, TX 77030, USA; 3 Department of Microbiology, Medical School, National and Kapodistrian University of Athens, 11527 Athens, Greece; cleoa@med.uoa.gr

**Keywords:** oropharyngeal microbiome, bacterial diversity, bacterial abundance, SARS-CoV-2 infection

## Abstract

Patients with COVID-19 infection have distinct oropharyngeal microbiota composition and diversity metrics according to disease severity. However, these findings are not consistent across the literature. We conducted a multicenter, prospective study in patients with COVID-19 requiring outpatient versus inpatient management to explore the microbial abundance of taxa at the phylum, family, genus, and species level, and we utilized alpha and beta diversity indices to further describe our findings. We collected oropharyngeal washing specimens at the time of study entry, which coincided with the COVID-19 diagnosis, to conduct all analyses. We included 43 patients in the study, of whom 16 were managed as outpatients and 27 required hospitalization. *Proteobacteria*, *Actinobacteria*, *Bacteroidetes*, *Saccharibacteria TM7*, *Fusobacteria*, and *Spirochaetes* were the most abundant phyla among patients, while 61 different families were detected, of which the *Streptococcaceae* and *Staphylococcaceae* families were the most predominant. A total of 132 microbial genera were detected, with *Streptococcus* being the predominant genus in outpatients, in contrast to hospitalized patients, in whom the *Staphylococcus* genus was predominant. LeFSe analysis identified 57 microbial species in the oropharyngeal washings of study participants that could discriminate the severity of symptoms of COVID-19 infections. Alpha diversity analysis did not reveal a difference in the abundance of bacterial species between the groups, but beta diversity analysis established distinct microbial communities between inpatients and outpatients. Our study provides information on the complex association between the oropharyngeal microbiota and SARS-CoV-2 infection. Although our study cannot establish causation, knowledge of specific taxonomic changes with increasing SARS-CoV-2 infection severity can provide us with novel clues for the prognostic classification of COVID-19 patients.

## 1. Introduction

The clinical spectrum of coronavirus disease 2019 (COVID-19) infection, caused by severe acute respiratory syndrome coronavirus 2 (SARS-CoV-2) virus, ranges significantly from asymptomatic disease and/or mild-to-moderate symptomatology to severe infection leading to acute respiratory distress syndrome (ARDS) and multi-organ failure [1]. Well-established risk factors that predispose an individual to severe disease include an advanced age, diabetes, cardiovascular disease, and obesity, among others [2,3,4]. Nevertheless, patients lacking classic risk factors for severe COVID-19 have also been found to exhibit symptomatology necessitating hospital admission and intensive management. Given the wide range of clinical manifestations of SARS-CoV-2 infection, considerable effort has been made to predict and explain the differential disease progression in individuals with COVID-19. Clinical manifestations and the severity of SARS-CoV-2 infection may be attributed to additional factors related to host–virus interactions, including the effect of the host microbiome on inflammatory responses elicited during viral infection [5].

The human respiratory tract microbiome is diverse and heterogeneous and is associated with a wide range of diseases and phenotypes. Microbial diversity is highest in the upper respiratory tract and decreases towards the lower respiratory tract. The upper respiratory tract provides a variety of niches for bacterial colonization, and the microbiome varies by location. The oropharyngeal microbiome modulates host immune responses against infectious agents by maintaining the physiologic microbiota of the respiratory tract and preventing the expansion of both commensal and opportunistic pathogens [6,7]. However, the respiratory microbiota diversity and composition can be profoundly altered in cases of severe pulmonary infectious insult, thus affecting the clinical course of the disease [8]. Regarding COVID-19, various studies have concluded that patients infected with the SARS-CoV-2 virus exhibit reduced upper respiratory tract microbial diversity and subsequent domination by pathogenic microbes, compared to healthy individuals [9,10]. The oral, nasal, and pharyngeal microbial profiles have all been studied in the literature, with results revealing various phyla, taxa, and species being affected in patients with COVID-19 [11]. Even more, the composition of the upper respiratory tract microbiome has been studied in patients with mild, moderate, and severe infection to gauge the impact of the microbiome on clinical outcomes and disease severity [12]. 

Although the literature is supported by studies that have found specific oropharyngeal microbial perturbations in patients with COVID-19, these associations are not consistent throughout the studies, mainly because of differences in sample collection methods, specimen analysis techniques, and the severity of symptoms of patients included in the studies [13]. As such, it is critical to further investigate the exact respiratory microbiota changes in patients with COVID-19, in order to consolidate existing findings and provide additional insight regarding the promising use of the microbiota as a prognostic indicator in patients with SARS-CoV-2 infection. The aim of our study was to assess the oropharyngeal microbiota composition and diversity in patients with SARS-CoV-2 infection requiring outpatient versus inpatient management.

## 2. Materials and Methods

### 2.1. Study Design

We conducted a multicenter prospective study, including patients ≥ 18 years of age residing in Northern Greece who were diagnosed with SARS-CoV-2 virus infection via laboratory confirmation, from 14 July 2021 until 17 November 2022. Patients were treated for COVID-19 in an outpatient setting or were hospitalized for further management. This study was approved by the Institutional Review Board of the School of Medicine of Aristotle University of Thessaloniki (decision No. 4509-22/02/2022). All patients provided written informed consent in accordance with the Helsinki Declaration and were subsequently enrolled in the study. Oropharyngeal washings were obtained from the patients at the time of study entry. The main outcome of the study was to investigate the association between microbial composition at the phylum, taxon, genus, and species level of the oropharyngeal microbiome and the severity of SARS-CoV-2 infection (i.e., outpatient management vs. hospitalization). Secondary outcomes included the association of the oropharyngeal microbial diversity (measured with the alpha- and beta-diversity indices) in patients with varying symptom severity (i.e., outpatient management vs. hospitalization).

### 2.2. Study Population

We included consecutive patients that were at least 18-years-old who were diagnosed with SARS-CoV-2 infection via laboratory confirmation. Specifically, we harnessed real-time reverse transcription polymerase chain reaction (rRT-PCR) using the TaqPath™ COVID-19 CE-IVD RT-PCR Kit (Applied Biosystem) for identifying open-reading frame (ORF) 1a/b (ORF1a/b), spike (S) protein, and N nucleocapsid N framework genes, which characterize SARS-CoV-2 virus infections. 

Patients were divided into two groups based on the severity of symptoms they exhibited at the time of SARS-CoV-2 diagnosis and disposition determined by the treating physician. These included patients with mild symptoms (including runny nose, cough, sore throat, myalgia, dysgeusia, anosmia, weakness, and fatigue) that were managed on an outpatient basis and patients with symptoms necessitating hospital admission for further treatment. The decision for hospital admission was based on the physician’s judgement and took into consideration the severity of symptoms experienced by the patient and underlying medical comorbidities. We limited the candidate patient pool to individuals residing in Northern Greece in an effort to reduce the effects of dietary and environmental factors on the host oropharyngeal microbiome composition and diversity. We excluded patients from the study who were receiving antibiotics at the time of enrollment or within 12 weeks prior to study initiation in an effort to reduce the impact of antibiotic therapy on the oropharyngeal microbiome composition and diversity.

### 2.3. Oropharyngeal Specimen Analysis

Molecular analysis of the oropharyngeal washings obtained from study participants was performed using Ion Torrent Ion sequencing technology and the Ion 16STM Metagenomics Kit (Thermo Scientific, Waltham, MA, USA). The kit includes 2 sets of primers, V2-4-8 and V3-6,7-9, which were used to amplify the corresponding hypervariable regions of the 16S gene in bacteria. The amplified fragments were sequenced on the Ion PGMTM platform and analyzed using the Ion 16STM metagenomics analyses module of Ion Reporter Software. The extraction of the genetic material from the oropharyngeal lavage of the patients was performed using the magnetic bead method on the ZYBIO EXM3000 machine, using the corresponding Zybio kit (Nucleic Acid Extraction kit–Magnetic Bead Method), according to the manufacturer’s instructions. 

### 2.4. Statistical Analysis

The FastQC program was used to check the quality of the sequenced reads. The reads were then filtered (trimmed) with IMNGS-toolbox [14], resulting in the exclusion of one sample due to the small sequencing depth. After filtering, the raw sequence reads, as FastQC files, were submitted to the Integrated Microbial Next Generation Sequencing (IMNGS) web platform [15], which uniformly and systematically screens, processes, and analyzes all available sets of prokaryotic 16rRNA to study and evaluate the distribution and diversity of microbial species colonizing the pharynx of patients with a COVID-19 infection. The files obtained from the IMNGS platform were then processed using the open source programming language R version 4.0.3 and the Rhea program [16] in order to study the alpha and beta diversity of the microbial populations colonizing the pharynx of patients with SARS-CoV-2 virus infection, as well as for their taxonomic characterization and a comparison between different groups of patients (e.g., patients requiring outpatient vs. inpatient management) to find possible correlations between specific microbial populations and the above groups. For alpha diversity, we harnessed the Chao1, Shannon, Simpson, and Fisher indices. For alpha diversity, we also calculated the effective diversity. The effective diversity is the number of equally abundant species that would give any value of a given index. When studying β-diversity in the Rhea program, a recent more balanced version of the generalized UniFrac [16] was used, and the visualization, in 2D space, of the distance matrix was performed using their Multi-Dimensional Scaling (MDS) and their non-metric version (NMDS), while the Calinski–Harabasz index was used to calculate the optimal number of groups. PERMANOVA analysis was applied (vegan:adonis software package, version 2.6-4) to determine whether the discrimination of groups was statistically significant (statistical significance level *p*-value = 0.05).

The EZBioCloud platform was used to identify microbial species down to the taxonomic species level [17]. To assess the magnitude of the effect of different microbial populations detected in the pharyngeal microbiome of patients with COVID-19 infection on the manifestation of different disease states, including classes (hospitalization–outpatient management, male–female, presence–absence of S gene), in addition to Rhea, Linear discriminant effect size analysis (LEfSe) was used in which the Kruskal–Wallis nonparametric test and multiple pairwise Wilcoxon tests were applied to generate a list of traits (microbial species) that can discriminate between classes [18].

## 3. Results

### 3.1. Patient Characteristics

We recruited 43 patients in the study, of whom 16 (37.2%) were managed in an outpatient setting, and 27 (62.8%) required hospitalization. The mean age of the patients was 58.2 years (range: 11–93 years) and 28 out of 43 (65.1%) were male. Hospitalized patients with COVID-19 were older than patients with COVID-19 managed on an outpatient basis (70 ± 18.7 vs. 39.1 ± 21.3, *p* < 0.05) (Table 1).

### 3.2. Abundance Analysis Results

A total of 8,116,768 reads were generated on the Ion Torrent platform, and after quality control with the FastQC program and filtering of the reads with the IMNGS-toolbox, one sample was excluded due to small sequencing depth. As such, the number of patients managed on an outpatient basis that were included in the abundance analysis was 15 instead of 16.

**Figure 1:**
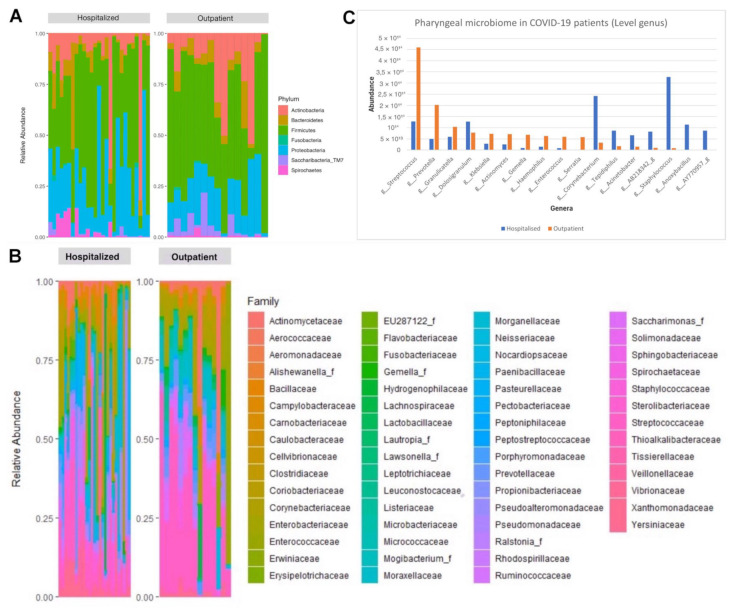
Relative abundance of dominant phyla (**A**), families (**B**), and genera (**C**) in patients with COVID-19 requiring hospitalization vs. patients treated on an outpatient basis.

A total of seven phyla were detected in the oropharyngeal washings of the patients, with *Firmicutes* (41.2%) being the most abundant, followed by *Proteobacteria* (28.4%), *Actinobacteria* (21.5%), *Bacteroidetes* (4.7%), *Saccharibacteria TM7* (2.8%), *Fusobacteria* (1.2%), and *Spirochaetes* (0.2%). At the family level, 61 families of microbial species were detected, and differences in the composition of the pharyngeal microbiome were observed in the study groups, with the *Streptococcaceae* and *Staphylococcaceae* families of the *Firmicutes* phylum being the most frequent and at the same time the most abundant families in both groups of patients. At the taxonomic genus level, 132 microbial genera were detected, with *Streptococcus* being the predominant microbial genus in patients with mild symptoms treated on an outpatient basis and in patients with the Delta (S+) coronavirus variant, in contrast to patients hospitalized with COVID-19 and patients with the Omicron (S−) coronavirus variant, in whom the *Staphylococcus* genus was predominant. Finally, at the taxonomic species level, 389 species were identified using the EZBioCloud platform, with *Streptococcus oralis* being the most abundant and constituting 10.52% of the total pharyngeal microbiome at the species level. In Supplementary Figure S1, we present the relative abundance of dominant taxa in the samples of patients included in the analysis. In Table 2, we present the number of phyla, families, genera, and species in the oropharyngeal microbiome of the study participants. In Figure 1, we present the relative abundance of dominant phyla, families, and genera in patients hospitalized with symptoms of COVID-19 vs. patients treated on an outpatient basis.

**Figure 2:**
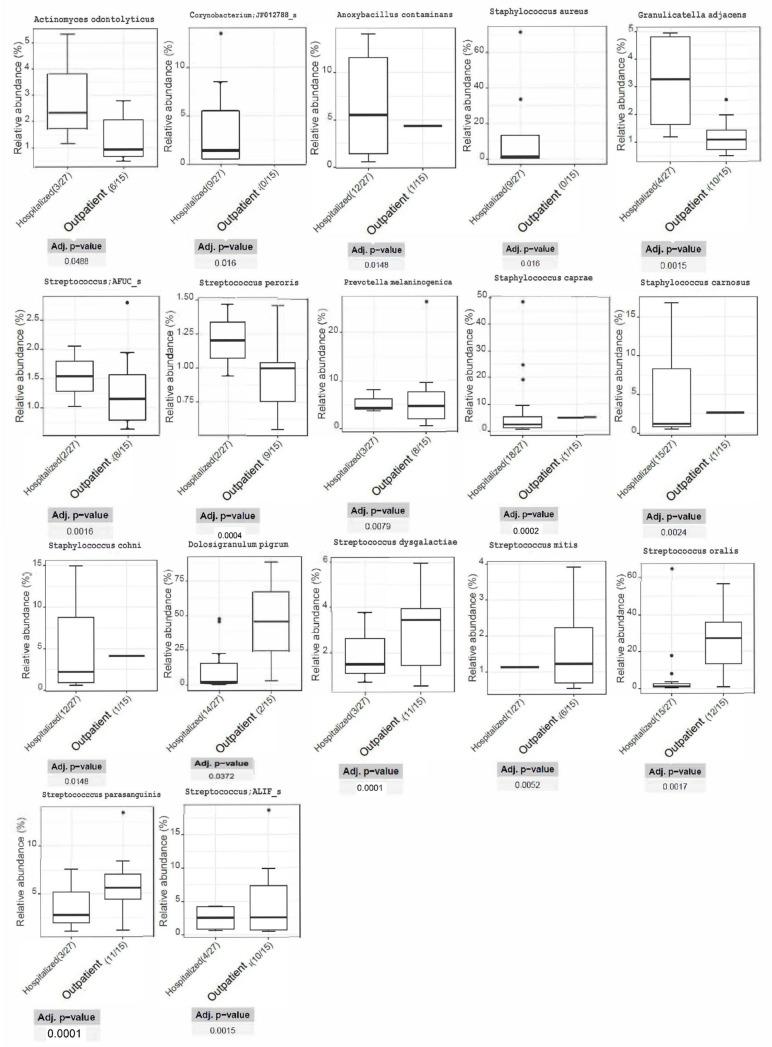
Relative abundance of dominant species in patients with COVID-19 requiring hospitalization vs. patients treated on an outpatient basis. Only statistically significant differences in the relative abundance of species are depicted. Note: *Streptococcus AFUC_s* and *Streptococcus ALIF_s* correspond to the *Streptococcus sinensis* group and *Streptococcus salivarius* group, respectively.

In Figure 2, we present the relative abundance of dominant species in patients that were hospitalized vs. patients that were treated as outpatients. Species from the genera *Staphylococcus*, *Streptococcus*, *Prevotella*, and *Dolosigranulum* were more abundant in hospitalized patients than in patients treated on an outpatient basis. Accordingly, species from the genera *Streptococcus*, *Corynebacterium*, *Actinomyces*, *Anoxybacillus*, and *Staphylococcus* were more abundant in outpatients than in patients with COVID-19 requiring hospitalization. In Supplementary Figure S2, we also present the genera of the *Proteobacteria*, *Actinobacteria*, *Firmicutes*, and *Bacteroidetes* phyla, which were the four most common phyla in our abundance analysis.

### 3.3. Alpha and Beta Diversity Analysis Results

**Figure 3:**
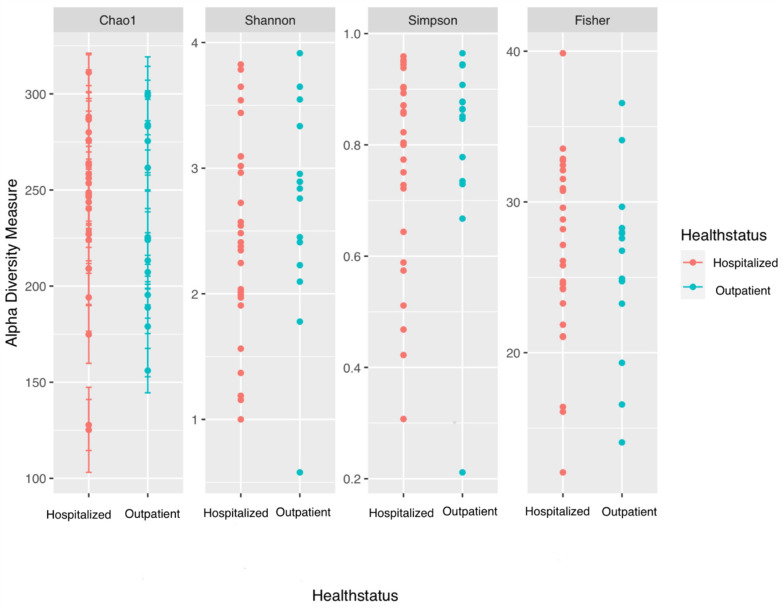
Alpha diversity analysis using the Chao1, Shannon, Simpson, and Fisher indices to evaluate comparative bacterial richness between patients with COVID-19 requiring hospitalization and patients treated on an outpatient basis.

**Figure 4:**
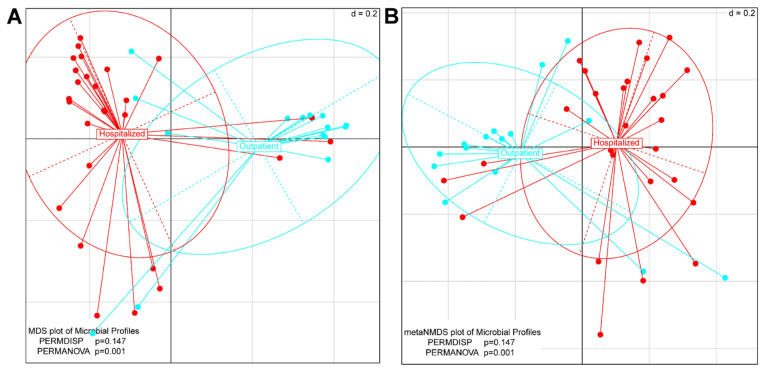
Visualization of β-diversity analysis using the Multi-Dimensional Scaling (MDS) (**A**) and the respective non-metric version (metaNMDS) (**B**).

Alpha diversity analysis did not reveal any statistically significant differences in the gender of patients (Supplementary Figure S3) with regards to bacterial species richness. Also, alpha diversity analysis with the Chao1, Shannon, Simpson, and Fisher indices did not find any statistically significant differences with regards to bacterial species richness between hospitalized patients with COVID-19 and patients with COVID-19 requiring outpatient management (Figure 3, Supplementary Table S1). When studying the beta-diversity of the microbial populations that composed the pharyngeal microbiomes of study participants, it was found that gender as a trait could not be used to distinguish the microbial profiles of the study patients (PERMANOVA, *p*-value = 0.329) (Supplementary Figure S4), as opposed to the severity of COVID-19 infection symptoms (outpatient management vs. hospitalization) (Figure 4).

### 3.4. LEfSe Analysis Results

**Figure 5:**
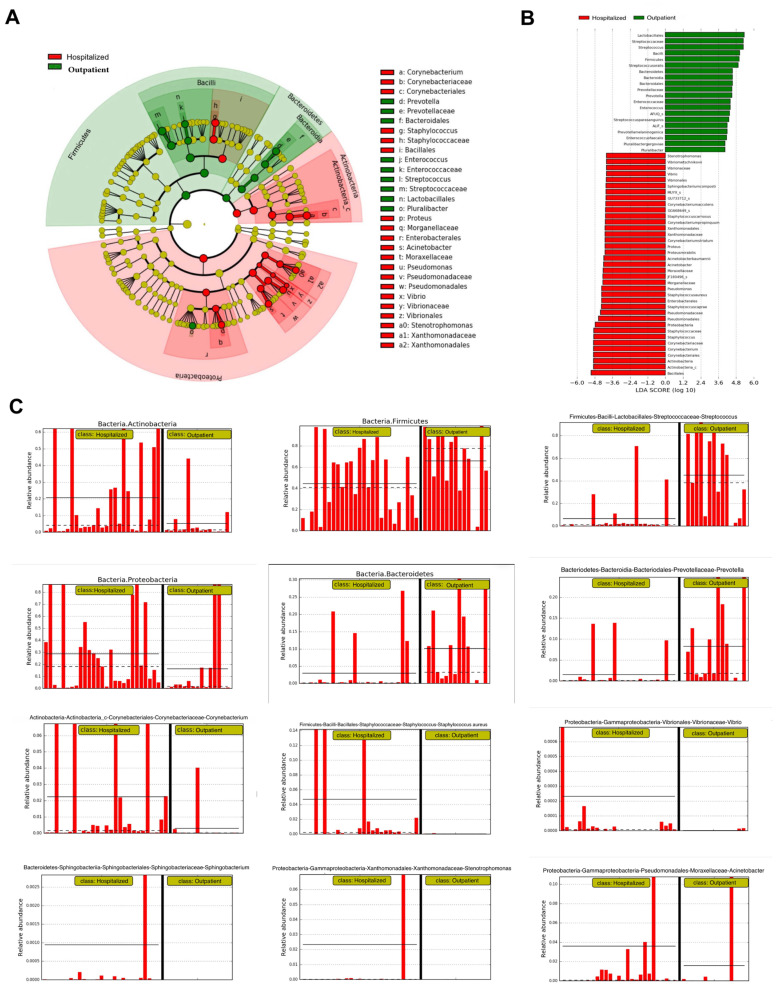
(**A**) Cladogram and (**B**) LDA score of the microbial species in the pharyngeal microbiomes of study patients, based on which the patients could be classified as those requiring inpatient management and those with mild symptoms treated on an outpatient basis. (**C**) Visualization of LEfSe analysis in the form of a bar chart of the main microbial species that could help to distinguish hospitalized patients with COVID-19 from patients with mild symptoms requiring treatment on an outpatient basis. The dotted and continuous horizontal lines indicate the median and mean values of each group, respectively.

To more specifically identify bacterial species associated with varying COVID-19 severity, LEfSe analysis was used to identify bacteria that differed significantly between groups. In this study, an LDA score >4 was used as the cut-off value. The cladogram representing the oropharyngeal microbial structure and the predominant bacteria identified 57 microbial species in the oropharyngeal washings of study participants that could discriminate the severity of symptoms of COVID-19 infection. Out of the 57 differentially abundant microbial species, 20 were remarkably increased in the group of patients treated as outpatients compared to inpatients, and 37 were increased in the group of patients with COVID-19 requiring hospitalization compared to outpatients (Figure 5A,B). In Figure 5C, we visualize the LefSe analysis results in the form of a bar chart to depict the main microbial species that could help distinguish hospitalized patients with COVID-19 from patients treated on an outpatient basis.

## 4. Discussion

The oropharyngeal microbiome has been studied in patients with varying COVID-19 severity, with results, however, revealing alterations in the microbiota composition and diversity that are not consistent across the studies. In our analysis, we revealed that bacterial richness was not significantly different between patients with COVID-19 treated as outpatients and hospitalized patients with SARS-CoV-2 infection. Beta diversity analysis revealed the distinct clustering of microbial communities in patients treated on an outpatient basis versus hospitalized patients with COVID-19. *Firmicutes* and *Proteobacteria* were the most abundant phyla in the hospitalized patients with COVID-19, which is in line with the published literature [6,19]. Also, LEfSe analysis revealed 57 differentially abundant microbial species that were either significantly enriched or reduced in patients with mild symptoms versus patients requiring hospitalization.

In our analysis, no community-level differences were observed in the oropharyngeal microbiomes of patients that were treated as outpatients versus inpatients. Consistent with our findings, Kim et al. [20] collected salivary and oropharyngeal samples from 60 hospitalized patients with COVID-19 and found that compositional differences in microbial communities between patients with severe symptoms requiring ICU admission versus those who did not were not significantly different. Merenstein et al. [21], however, examined 507 oropharyngeal, nasopharyngeal, and endotracheal samples from 83 hospitalized patients of varying severity and found significant differences in alpha-diversity analysis results between patients of varying disease severity. In alignment with the latter study, the majority of studies published in the literature have found a negative correlation between alpha diversity and SARS-CoV-2 infection severity [22,23,24]. However, other studies have concluded that SARS-CoV-2 infection does not significantly impact the oropharyngeal and respiratory microbiota composition [25,26]. The failure of our analysis to reveal significant associations between alpha diversity and symptom severity may be attributed to the small sample size, discrepancies in the definition of COVID-19 severity, and varying sampling site locations across the upper airway respiratory tract, compared to previous analyses. Since the majority of data stem from studies comparing patients with COVID-19 versus healthy controls, it is important to conduct additional analyses in patients with varying COVID-19 severity to establish distinct microbial community perturbations in patients with SARS-CoV-2 infection stratified by the symptom severity and disposition status.

The relative abundance of *Corynebacterium* and *Staphylococcus* was significantly higher in hospitalized patients with COVID-19 than in outpatients in our study. Similar findings were obtained by Hernández-Terán et al. [27] who revealed that the abundance of both *Staphylococcus* and *Corynebacterium* genera was increased in the respiratory tract of patients with severe compared to mild COVID-19 infection. Kumar et al. [28] also showed that the abundance of *Corynebacterium* and *Staphylococcus* was increased in patients with COVID-19 who died compared to patients who recovered. However, Yasir et al. [29] and Mostafa et al. [30] found a decrease in the relative abundance of *Corynebacterium* with an increasing severity of COVID-19 infection. A study by Szabo et al. [31] revealed that *Corynebacterium* spp., especially *C. accolens*, showed significantly higher abundance in the nasopharynx of uninfected individuals versus patients with COVID-19, with the downregulation of angiotensin-converting enzyme 2 (ACE-2) and transmembrane serine protease 2 (TMPRSS2) and inhibition of S1–ACE-2 binding being proposed as the main mechanisms of reduced susceptibility to infection in patients who harbor *Corynebacterium* spp. These discordant findings may be attributed to specific changes in the relative abundances of various species within the *Corynebacterium* genus, highlighting the need for analyses at the species level to delineate the abundance trends of the aforementioned genera. 

In our study, the relative abundance of *Streptococcus, Prevotella*, and *Actinomyces* was higher in outpatients than in hospitalized patients. The relative abundance of the majority of *Streptococcus* genera in our study was significantly higher in hospitalized patients than in outpatients. Ma et al. performed a metagenomic analysis of the oropharyngeal microbiomes of SARS-CoV-2-infected individuals and healthy controls and found that the relative abundance of *Streptococcus* was higher in healthy individuals. However, Hernández-Terán et al. [27] characterized the respiratory microbiota composition of COVID-19 patients with mild, severe, and fatal infection and found a gradual increase in the abundance of the *Streptococcus* genus with increasing symptom severity. Next, the higher relative abundance of *Prevotella* in outpatients is in line with published studies that describe higher abundance in patients with mild compared to severe COVID-19 symptoms. Ventero et al. [32] and Bradley et al. [33] have found a negative correlation between the relative abundance of *Prevotella* and SARS-CoV-2 infection severity. *Prevotella* species constitute a component of the endogenous airway microbiota, and studies have revealed that an increasing abundance of such species confers protection against bacterial pathogens [34]. However, Lu et al. [35] concluded that the relative abundance of *Prevotella* was higher in SARS-CoV-2-infected individuals than in healthy controls and proposed that *Prevotella* can modulate sphingolipid metabolic pathways that promote viral replication and subsequent inflammation. Finally in our study, the relative abundance of *Actinomyces* was found to be significantly higher in the group of patients requiring outpatient management than in hospitalized patients. Bourumeau et al. [19] assessed the oropharyngeal microbial profile of 182 COVID-19 patients compared to 75 healthy controls and found *Actinomyces* to be a marker of SARS-CoV-2-negative individuals. Li et al. [10] processed samples from COVID-19 patients and healthy controls and found *Actinomyces* to be significantly enriched in COVID-19 patients. The discrepancies in the abundance analysis results between the published studies among the aforementioned genera underlines the importance of conducting further research to elucidate the specific species of the above genera that contribute to COVID-19 pathogenesis and symptom severity.

Our study has limitations that need to be acknowledged. First, our sample size was small, and this might explain the lack of significant findings with regards to alpha diversity analysis results and the relative abundance of various taxa reported to be significantly altered in other studies. Unfortunately, further recruitment of study participants was not feasible due to funding issues. Next, underlying patient comorbidities were not available in our analysis, and, as such, we could not comment on whether they could have affected the oropharyngeal microbiota composition. Also, information regarding preventive practices, such as the use of face masks and/or alcohol or non-alcohol-based oral rinses and mouth washes, were not available for the patients included in our study. Even more, for patients admitted to the hospital, information about a higher level of care (i.e., ICU admission, endotracheal intubation, and mechanical ventilation) was not available, which prevented us from carrying out additional analyses of patients hospitalized for the management of COVID-19. Also, the age difference between inpatients and outpatients was statistically significant, and since advancing age is associated with changes in the microbiota composition of the human body, no causal link can be established between our findings and the COVID-19 severity determination. It needs to be emphasized that correlation does not imply causality, and some common factors between inpatients and outpatients that were not accounted for could affect the composition of the microbiome and the host response to SARS-CoV-2 infection and subsequent severity of the disease. Although antimicrobial therapy was an exclusion criterion in our study, other treatment regimens were not documented for patients included in the analysis, and we could not assess whether the two groups had significant differences with regards to underlying medications that could affect the microbiota composition. Finally, although patients were recruited from a distinct regional compartment of Greece in order to mitigate the effects of residence and diet on the oropharyngeal microbiota composition, other unknown confounders that were not accounted for may have affected our analyses.

## 5. Conclusions

In conclusion, our study provides information on the complex association between the oropharyngeal microbiota and SARS-CoV-2 infection. Although our study cannot establish causation, knowledge of specific taxonomic changes with increasing SARS-CoV-2 infection severity can provide us with novel clues for the prognostic classification of COVID-19 patients. Well-designed, prospective studies are required to ascertain the impact of the oropharyngeal microbiome in patients with varying severity of SARS-CoV-2 infection and assess whether distinct microbial community assemblies could risk-stratify patients with COVID-19.

## Figures and Tables

**Table 1:** Demographic data of patients included in the study.

	Hospitalized Patients with COVID-19	Patients with COVID-19 Managed as Outpatients
Subjects, N	27	16
Age, years ± SD (range)	70 ± 18.7 (30–93)	39.1 ± 21.3 (11–76)
Male: Female	18/9	10/6

Abbreviations: COVID-19: Coronavirus disease 2019; N: number; SD: standard deviation.

**Table 2:** Number of families, genera, and species according to phylum in the pharyngeal microbiomes of patients included in the study.

Phylum	Family, N	Genus, N	Species, N
*Firmicutes*	20	49	154
*Proteobacteria*	25	59	116
*Actinobacteria*	8	11	58
*Bacteroidetes*	4	8	45
*Saccharibacteria TM7*	1	2	7
*Fusobacteria*	2	2	6
*Spirochaetes*	1	1	3

Abbreviations: N: number.

## Data Availability

Data for this manuscript are available and can be provided upon reason request.

## Supplementary Materials

The following supporting information can be downloaded at: https://www.mdpi.com/article/10.3390/jpm14040369/s1, Figure S1: Relative abundance (%) of dominant taxa (at the phylum, class, family, and species level) in samples of patients included in the analysis; Figure S2: Genera of Firmicutes (A), Proteobacteria (B), Actinobacteria (C), and Bacteroidetes (D) in patients (hospitalized versus outpatients) included in the study; Figure S3: Alpha diversity analysis using the Chao1, Shannon, Simpson, and Fisher indices to evaluate comparative bacterial richness between female and male patients with COVID-19; Figure S4: Multi-dimensional scaling for the pharyngeal microbiome of the study patients based on the gender of the patients included in the analysis; Table S1: Evaluation of alpha-diversity analysis for the samples included in the analysis.
