# Supplementary material for: Oropharyngeal Microbiome Analysis in Patients with Varying SARS-CoV-2 Infection Severity: A Prospective Cohort Study
Source: J Pers Med. 2024 Mar 29;14(4):369. doi: 10.3390/jpm14040369 (PMC11051038; doi:10.3390/jpm14040369)
Supplement: Supplementary file 1 [file jpm-14-00369-s001.zip › Supplement/JPM-Supplementary Appendix-03.14.2024.pdf]

Supplement to: Oropharyngeal Microbiome Analysis in Patients with Varying SARS-CoV-2 Infection Severity: A Prospective Cohort Study

Contents

Section 1: Supplemental Tables and Figures

Table S1: Evaluation of alpha-diversity analysis for the samples included in the analysis.

Figure S1. Relative abundance (%) of dominant taxa (at the phylum, class, family, and species level) in samples of patients included in the analysis

Figure S2: Genera of *Firmicutes* **(A)**, *Proteobacteria* **(B)**, *Actinobacteria* **(C)**, and *Bacteroidetes* **(D)** in patients (hospitalized versus outpatients) included in the study.

Figure S3: Alpha diversity analysis using the Chao1, Shannon, Simpson, and Fisher indices to evaluate comparative bacterial richness between female and male patients with COVID-19.

Figure S4: Multi-dimensional scaling for the pharyngeal microbiome of the study patients based on the gender of the patients included in the analysis.

Table S1: Evaluation of alpha-diversity analysis for the samples included in the analysis.

Sample	Condition	Richness	Normalized Richness	Effective Richness	Shannon Index	Shannon Effective	Simpson Index	Simpson Effective	Evenness
1_1189	Hospitalized	139	94	46	364.760.142.812.581	38.38	0.047600972	21.01	0.51238
10_1061	Hospitalized	71	40	19	20.365.134.183.661	7.66	0.272160609	3.67	0.331154
11_203	Hospitalized	30	20	13	198.965.801.864.762	7.31	0.177329507	5.64	0.405482
12_906	Hospitalized	78	48	19	224.531.544.254.865	9.44	0.19988135	5	0.357227
13_1382	Hospitalized	31	13	9	136.994.496.874.746	3.94	0.356313423	2.81	0.276522
14_1770	Hospitalized	48	25	14	118.838.973.141.582	3.28	0.577736988	1.73	0.212784
15_65	Hospitalized	75	23	8	115.496.416.412.524	3.17	0.53183034	1.88	0.185423
16_68	Hospitalized	79	43	18	201.224.211.984.982	7.48	0.27827687	3.59	0.319212
17_443	Outpatient	37	21	6	0.578236931	1.78	0.788599663	1.27	0.110998
18_981	Outpatient	113	69	29	222.756.483.769.007	9.28	0.332550646	3.01	0.326614
19_982	Outpatient	153	108	51	364.801.286.992.949	38.4	0.056802958	17.6	0.502662
2_1431	Hospitalized	129	88	62	378.481.525.637.569	44.03	0.040929132	24.43	0.539822
20_1034	Outpatient	115	77	31	241.028.329.681.219	11.14	0.265176219	3.77	0.352098
21_1118	Outpatient	93	56	30	245.112.612.685.133	11.6	0.221940802	4.51	0.374838
22_89	Hospitalized	131	87	44	34.401.771.474.737	31.19	0.061525437	16.25	0.489118
23_160	Hospitalized	129	88	45	354.048.604.596.836	34.48	0.050898223	19.65	0.504974
24_180	Hospitalized	132	78	40	309.376.879.538.643	22.06	0.098375909	10.17	0.439182
25_181	Hospitalized	139	83	32	301.688.335.085.924	20.43	0.107127322	9.33	0.423783
26_224	Outpatient	169	111	57	39.146.802.524.318	50.13	0.035312567	28.32	0.528948
27_989	Outpatient	120	85	39	295.452.546.876.936	19.19	0.122261694	8.18	0.427765

28_1116	Outpatient	129	91	33	283.738.969.065.517	17.07	0.14865261	6.73	0.404692
29_143	Outpatient	120	87	46	333.443.733.156.452	28.06	0.092240952	10.84	0.48277
3_1765	Outpatient	39	21	12	209.640.739.326.217	8.14	0.152916719	6.54	0.396641
30_383	Outpatient	105	74	37	289.191.238.443.877	18.03	0.123190453	8.12	0.430713
32_1206	Outpatient	124	82	40	289.165.236.611.308	18.02	0.136621385	7.32	0.415814
33_180	Hospitalized	125	69	32	296.349.199.150.989	19.37	0.096494019	10.36	0.425436
34_181	Hospitalized	79	47	23	25.709.552.796.877	13.08	0.128853231	7.76	0.407843
35_182	Hospitalized	160	77	45	309.358.159.211.222	22.06	0.09605147	10.41	0.422509
36_186	Hospitalized	107	61	34	254.253.026.573.201	12.71	0.226397581	4.42	0.377148
37_189	Hospitalized	92	62	30	190.692.545.779.767	6.73	0.425904932	2.35	0.292314
38_199	Hospitalized	126	66	26	196.979.768.186.397	7.17	0.411432391	2.43	0.282316
39_201	Hospitalized	109	65	33	240.983.803.592.655	11.13	0.24932376	4.01	0.356054
4_1082	Hospitalized	44	22	17	234.599.091.113.819	10.44	0.140527001	7.12	0.429713
40_216	Hospitalized	97	59	23	237.692.346.581.775	10.77	0.195879651	5.11	0.360145
41_75	Hospitalized	98	39	14	100.001.062.763.204	2.72	0.692592574	1.44	0.15118
42_991	Outpatient	91	52	31	275.833.654.518.053	15.77	0.135688995	7.37	0.423851
43_151	Hospitalized	92	57	34	272.379.474.723.361	15.24	0.143708933	6.96	0.417532
5_1044	Outpatient	34	20	11	177.836.308.870.424	5.92	0.270451249	3.7	0.349558
6_1094	Hospitalized	185	134	58	382.557.706.197.161	45.86	0.055646224	17.97	0.507952
7_204	Hospitalized	72	35	19	248.278.769.554.005	11.97	0.129407957	7.73	0.402402
8_670	Outpatient	112	83	54	354.687.832.379.835	34.7	0.055329624	18.07	0.521036
9_905	Hospitalized	50	36	22	156.291.686.688.353	4.77	0.488684737	2.05	0.276924

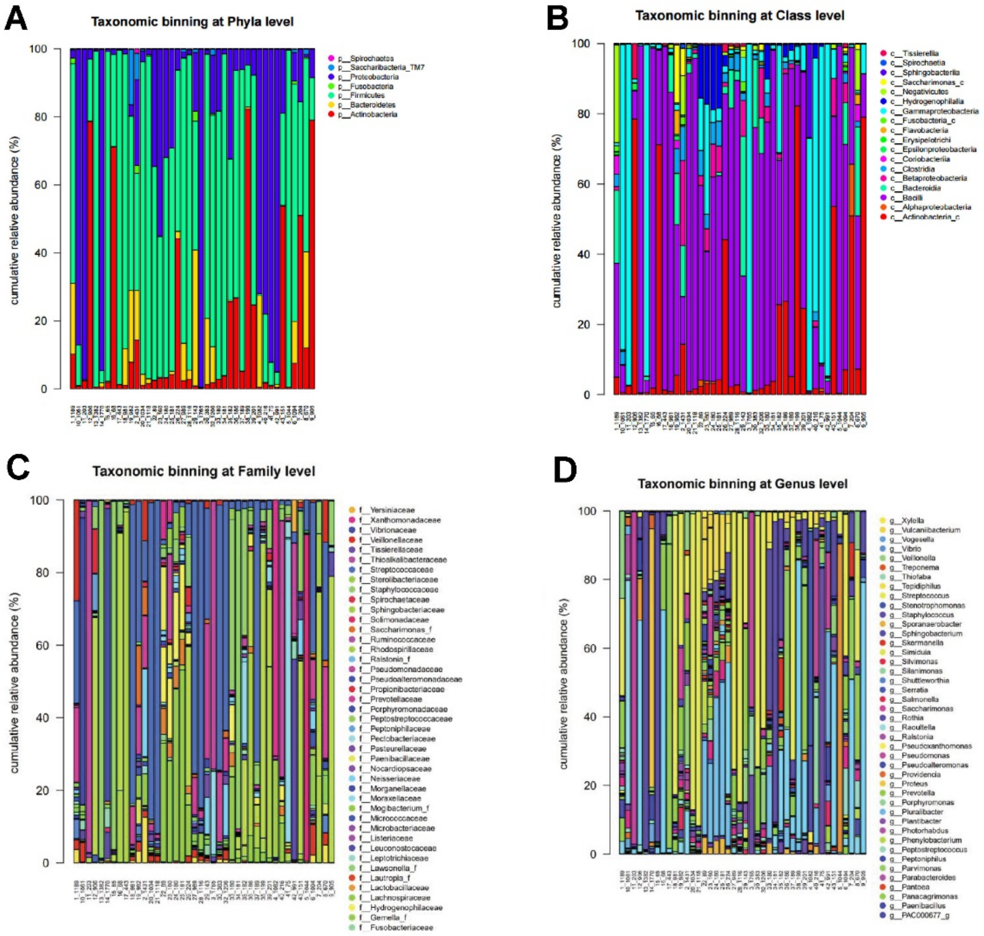

Figure S1. Relative abundance (%) of dominant taxa (at the phylum, class, family, and species level) in samples of patients included in the analysis.

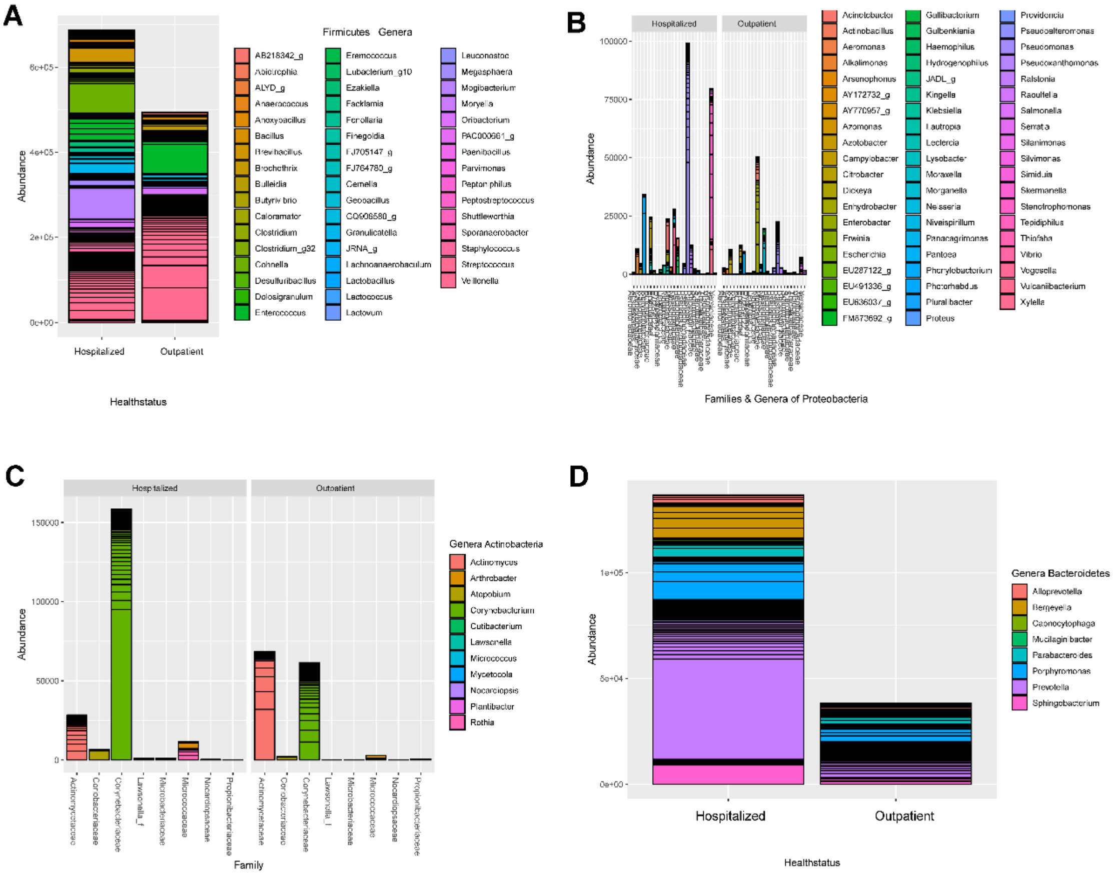

Figure S2: Genera of *Firmicutes* **(A)**, *Proteobacteria* **(B)**, *Actinobacteria* **(C)**, and *Bacteroidetes* **(D)** in patients (hospitalized versus outpatients) included in the study.

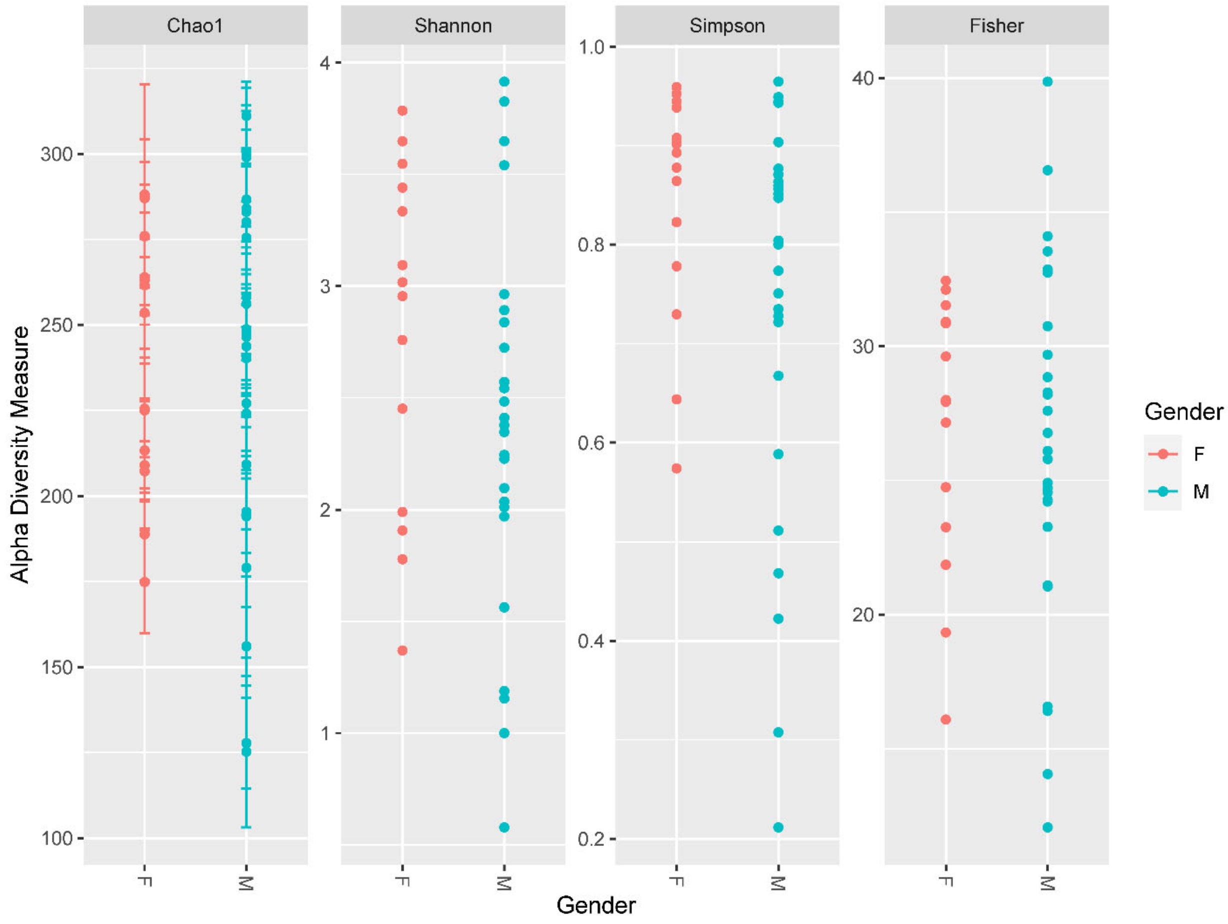

Figure S3: Alpha diversity analysis using the Chao1, Shannon, Simpson, and Fisher indices to evaluate comparative bacterial richness between female and male patients with COVID-19.

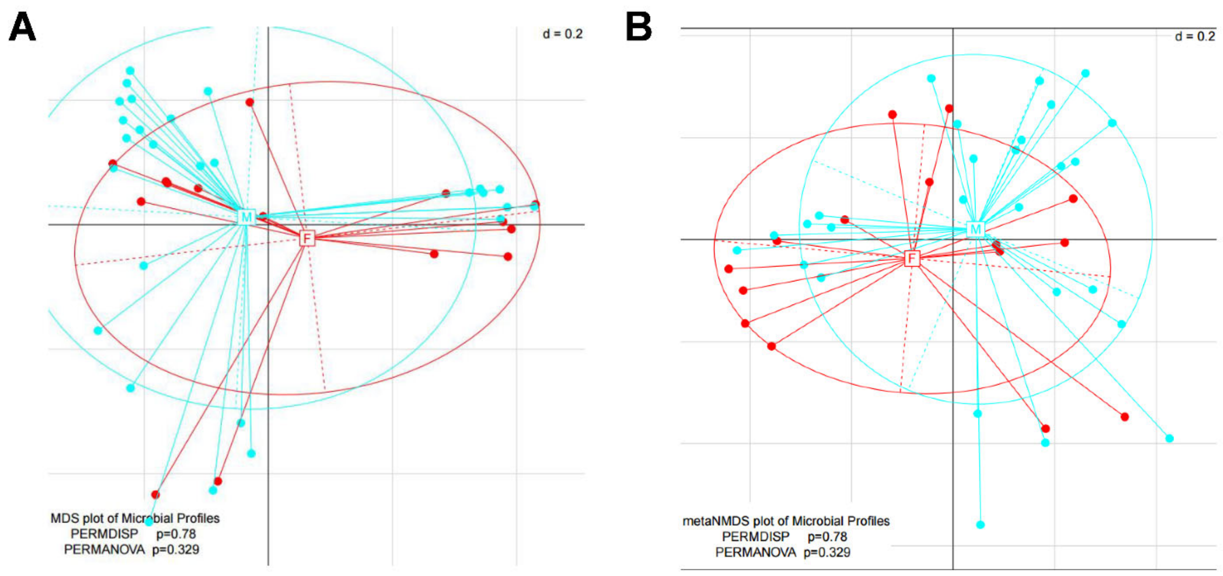

Figure S4: Multi-dimensional scaling for the pharyngeal microbiome of the study patients based on the gender of the patients included in the analysis.
